# Rapid human subjects research in times of disruption

**DOI:** 10.3389/fsoc.2022.959730

**Published:** 2022-09-16

**Authors:** Chelsea A. LeNoble, Kristin A. Horan, Nina Steigerwald

**Affiliations:** ^1^Embry-Riddle Aeronautical University, Daytona Beach, FL, United States; ^2^Department of Psychology, University of Central Florida, Orlando, FL, United States; ^3^Department of Psychological Science, Kennesaw State University, Kennesaw, GA, United States

**Keywords:** research methods, rapid research, human subjects, COVID-19, social science

## Abstract

One significant challenge facing the implementation of rapid research studies, or research that responds quickly to societal needs, involves the recruitment and retention of human subjects research participants. The purpose of this paper is to offer insights into the nuances of conducting rapid research during times of disruption. The first-hand accounts of participants experiencing disruption are critical and perishable. Although it may be difficult to recruit and retain participants, their data are needed to best understand and learn from novel, unprecedented situations. To this end, the authors draw from and analyze their experience conducting rapid research funded by the National Science Foundation to examine the effects of the COVID-19 pandemic on undergraduate education. The paper begins with a summary of the rapid project aims and research questions. Then, participant recruitment and retention challenges are briefly introduced as an advanced organizer of the paper. From there, the paper is structured in three sections that represent the human subjects research challenges faced during rapid study implementation. In the discussion, the authors summarize the above challenges and lessons learned in the larger context of rapid research. They reflect on a sometimes-forgotten issue: the wellbeing of research team members who face these and other salient challenges reflective of navigating life during a worldwide pandemic. By describing human subjects research challenges experienced in the implementation of a rapid study and lessons learned from experiencing and adapting to these challenges, this paper contributes meaningful insights into the daily challenges of carrying out rapid research.

## Introduction

In March 2020, the World Health Organization (WHO) declared the novel coronavirus disease 19 (COVID-19) a global pandemic following millions of confirmed cases and deaths worldwide (Johns Hopkins University, 2021). The pandemic significantly impacted higher education, and many U.S. institutions were forced to close their campuses, transition to online learning, restrict travel, and cancel professional conferences (Alexander, 2020; Gruber, 2020). These unprecedented changes greatly impacted teaching pedagogy and student learning. Considering the severe negative effect that natural disasters and pandemics can have on wellbeing (Main et al., 2011) as well as the disaster-related challenges institutions of higher learning are vulnerable to (Higher Education Information Security Council, n.d.), it became apparent that rapid research examining faculty and students' teaching and learning attitudes, perspectives, and behaviors during the COVID-19 pandemic was desperately needed.

The importance of capturing the experiences of those who live through COVID-19 disruption cannot be overstated; studying human behavior during disasters advances our understanding of social science phenomena (Reinhardt and Ross, 2019). However, human subjects research conducted during times of disruption and disaster is characterized by complexities that challenge our ability to not only conduct rigorous rapid research but also derive meaningful insights (Peek and Guikema, 2021). While a great deal of research has been conducted on the effects of disasters on human behavior and wellbeing [e.g., the severe acute respiratory syndrome epidemic (SARS) in China by Main et al., 2011 and Mihashi et al., 2009; the Ya'an earthquake by Wang et al., 2020; SARS and COVID-19 by Zhao et al., 2021], gaps remain in our understanding of the best ways to conduct human subjects research during major crisis and disaster events. For instance, as the pandemic has changed the research landscape so that face-to-face studies now rely on online data collection and remote collaboration (Clay, 2020), social science scholars must challenge assumptions about the ways that we recruit and engage with study participants.

The purpose of this article is to describe challenges faced by the authors' research team while conducting COVID-19 human subjects research and bring attention to important logistical issues that must be addressed by future research and research policy efforts alike. Leaders in the disaster research community have advised that “local researchers should conduct research on local disasters” (Oulahen et al., 2020, p. 570). As university professors and students navigating COVID-19 disruptions, each member of the research team was dedicated to contributing new knowledge to help institutions of higher education navigate the COVID-19 pandemic and future threats yet to come.

In what follows below, the authors of this article offer insights into the nuances of conducting human subjects rapid research from their own experience with a National Science Foundation funded study to examine the effects of the COVID-19 pandemic on undergraduate education. The insights from this experience are categorized into three themes: inter-institutional research team coordination, institutional recruitment, and participant retention.

### The rapid research study

With an emphasis on teaching and learning within undergraduate science, technology, engineering, and mathematics (STEM) education, the research project was designed to (1) examine teaching and learning experiences of undergraduate faculty and students in response to the COVID-19 pandemic, (2) examine undergraduate STEM teaching and learning impacts, and (3) leverage findings to develop recommendations for colleges and universities to best prepare and protect their faculty, staff, and students and the integrity of undergraduate STEM education in the future. The project was developed in response to the National Science Foundation (NSF) Dear Colleague Letter distributed in April, 2020 that encouraged the submission of COVID-19 rapid research proposals “having a severe urgency with regard to availability of or access to data, facilities or specialized equipment as well as quick-response research on natural or anthropogenic disasters and similar unanticipated events” (National Science Foundation, 2020).

The study fit the NSF conceptualization of rapid research for a few reasons. First, it was crucial to begin data collection of survey and interview responses as soon as possible given the fluctuating national milestones (lifting of stay-at-home orders and non-essential business closures) and general heightened sense of uncertainty characterizing higher education in the United States at this time (e.g., the status of graduation ceremonies, whether courses will resume online in summer and fall). Second, the context of continued disruption to normal modes of operating presented an important starting point for examining institutions' and individuals' responses to COVID-19. As national responses and education decisions continued to rapidly unfold, the opportunity to capture the nature of undergraduate education experiences during such a critical time of fluctuation and uncertainty was ephemeral. Finally, individuals are unlikely to accurately later recall the extent to which they were able to adapt to changes, the extent to which changes created distress, and how they coped with events; as a result, psychological research emphasizes the importance of real-time measurement of experiences, emotions, and behavioral reactions (Shiffman et al., 2008).

**Figure 1:**
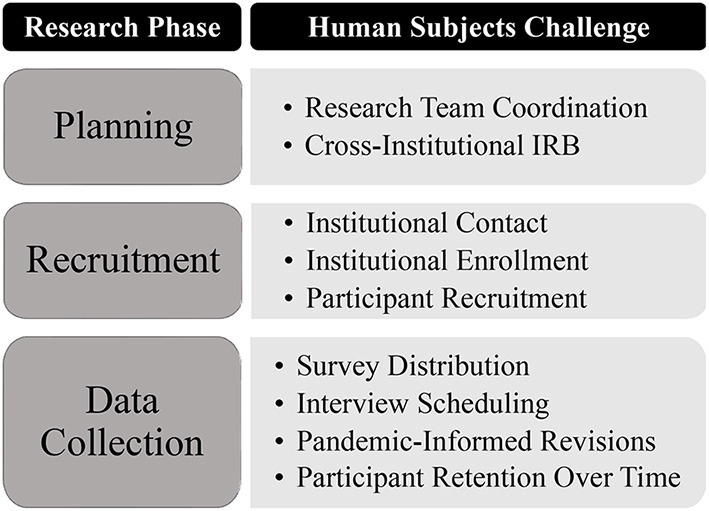
Human subjects challenges experienced across research phases.

Based on existing knowledge and gaps in the literature at the time, the study research questions focused on understanding the impacts of COVID-19 related institutional communication, transitions to online instruction, and COVID-19 resources on undergraduate faculty and student attitudes, perceptions, and behavior over time. To this end, a longitudinal, multi-method approach was used to gather information on institutional policy, crisis communication, and resulting attitudes, perceptions, and behaviors of ~400 faculty and 1,900 students from representative U.S. institutions across the Carnegie Basic Classification categories (The Carnegie Classification of Institutions of Higher, n.d). Data including archival responses from the Integrated Postsecondary Education Data System (IPEDS) (U.S. Department of Education, 2018), internet-based self-report surveys, semi-structured Zoom interviews, and over 4,000 messages sent from institutions to their faculty and students were collected at three points from the summer of 2020 to the spring of 2021. The challenges faced at each phase of the research project are summarized in Figure 1.

### Challenge 1: Inter-institutional research team coordination

The first human subjects rapid research challenge involved coordination occurring within the inter-institutional research team. While research collaborations across institutions create unique benefits such as a widened pool of research resources and expertise, they also pose challenges that have been documented by previous scholars (Bogue et al., 2005; Tigges et al., 2019). Issues such as navigating the particular policies and procedures of each institution, protecting human subjects data, and balancing other projects and priorities during a global pandemic all contributed complexity to the completion of the rapid research project.

The research team needed to design research procedures that accounted for the Institutional Review Board (IRB) requirements of each home institution. This involved having the research team members coordinate and communicate with one another and with their home institutions regarding policies and procedures for securing initial IRB approval, completing any necessary IRB modifications over the course of the project, and ensuring project objectives were completed as intended and as approved in by the IRB. For instance, it was important to efficiently communicate participation information to facilitate accurate and timely distribution of research incentives.

To adapt to these challenges, the research team followed recommendations developed in the science of team science (e.g., Bennett et al., 2018) to create a collaborative and communication-oriented structure. This involved coordinating meeting schedules across not only two different academic calendars but also two different calendar systems (16-week semesters vs. 9-week terms) to make sure that there was consistent information flow about all project needs, activities, and developments. The team held videoconference meetings every other week to convey project updates, resolve issues that arose, and come to consensus on any decisions that needed to be made or changes that needed to be implemented. Closed-loop communication was enacted so that even if a team member was not able to immediately address an email they received, they would reply to convey that they received the message and when they would be able to fully respond. For the survey component of the project, the research team maintained shared access to the survey using the collaborative function in the survey platform. Finally, a data preparation meeting was held once all survey data were collected. In this meeting, the team worked to download, organize, and clean the dataset, walking through multiple steps together so that everyone had a shared mental model of how the data were structured prior to splitting up analysis and reporting tasks.

Overall, this coordination was important to the research process because it helped to maintain a shared understanding of the ongoing research effort and keep research team members engaged over time. Especially in a time of global disruption and uncertainty, any infrastructure that can be implemented to promote and sustain collaborative processes will greatly benefit team science efforts broadly and rapid research efforts specifically. This includes infrastructure not just at the research team level, but also at the institution level. As recent work (Rohrbach and Genco, 2022) has noted, reward structures within institutions traditionally prioritize individual pursuits, often at the expense of team science and interdisciplinary collaborations. As such, there is an opportunity for institutions of higher education and the professional organizations of various disciplines to develop inter-institutional guidelines and processes that would better facilitate rapid research. A recent article by Peek et al. (2021) provides excellent recommendations regarding what institutions can do to facilitate interdisciplinary and inter-institutional rapid response research. One specific example is the development of an IRB Authorization Agreement between institutions that can “increase ethical standards, reduce the burden to participants, and streamline efforts to get well-trained researchers into the field rapidly when a disaster occurs” (Peek et al., 2021, p. 1210).

### Challenge 2: Institutional recruitment

A second challenge encountered by the research team was institutional recruitment. It was imperative to engage a diverse set of higher education institutions in the research project because the challenges faced by institutions and their capacity to respond likely varied according to certain characteristics of the institution. The research team employed modified stratified sampling to invite institutions to participate and aid in recruitment. Because participatory research practices strengthen community-based research efforts (Wallerstein et al., 2019), it was a goal of the research team to recruit individuals to the study from institutions that fully supported their participation. This involved working with institutions' IRB coordinators and administrators to ensure that research logistics led to distribution of study materials and participant recruitment aligned with their institution culture and mission.

In May 2020, the research team created a recruitment pool of higher education institutions. The 2018 Integrated Postsecondary Education Data System (IPEDS) database was referenced to create stratified samples ensuring accurate representation across institutional characteristics [e.g., public/private, urban/suburban/rural, small/medium/large, status as a Historically Black College or University (HBCU) or Hispanic Serving Institution (HSI), Carnegie Classification]. Approximately 90 institutions were invited in the first wave of university recruitment; in subsequent waves of recruitment, when a university did not respond or declined participation, another institution was randomly selected from the same strata (i.e., a small, urban, private, HBCU university). Ultimately, there were three waves of recruitment and approximately 690 universities were contacted.

Multiple professionals (ranging from university president to deans of faculty and student affairs) at each institution were contacted by email and phone. Still, acceptance was low. Although a technical report containing institution-specific data and recommendations was an incentive for partnering institutions, this did not seem to justify participation for many. The most common reason to decline participation was the institution perceived that faculty and students were already overburdened.

To overcome this challenge, the research team deployed a multifaceted approach: seeking alternative institutional contacts and pursuing institutional oversampling. Although the first waves of recruitment originally involved reaching out to higher levels of leadership within university administration—based on the idea that a message of support from leadership could increase buy-in among participants—non-response rates likely correlated with the level at which upper-level administrators were overburdened. Subsequent waves of recruitment involved contacting lower levels of administration and/or administrative assistants. This strategy earned a modest increase in the number of responses. It is also worth noting that the principal investigators virtually met with administrators upon request to provide more information and discuss recruitment or participation concerns.

The next facet of the approach to improving the participation rate was oversampling within each strata in subsequent waves of recruitment. This activity supplanted the original stratification strategy of replacing a selected institution who did not agree to participate with one randomly selected university of similar IPEDS characteristics. The research team identified the institution acceptance rate and oversampled at a rate that would likely produce the desired number of institutions even if the rate of nonresponse or declining participation remained consistent.

Although responses from institutions did increase at this stage, the research team did not achieve the desired institutional sample size of 90 institutions. Undergraduate students and faculty from 33 institutions participated in the study; these institutions were generally representative of the national characteristics identified from IPEDS. Finally, the researchers discussed modifications to the study design that could preserve the original intent of a large institutional sample size, allowing for examination of a breadth of COVID-19 responses. A team of trained research assistants obtained publicly available COVID-19-related messages from the universities that were originally randomly sampled from IPEDS to provide additional information about institutional responses to the pandemic.

In responding to these challenges, the research team gained the following insights. Rapid research conducted during times of crisis must anticipate the challenges faced by the organizations that they are attempting to serve. Oversampling can help a research team in gathering data quickly when rates of non-response will likely be present during difficult times. Additionally, the research team learned the value of considering alternatives to data collection methods that require human time and effort. Instead of solely depending on participant recall, publicly available data could be gathered that reflected the response of a large, representative sample of institutions.

### Challenge 3: Participant retention

The final rapid research challenge involved the retention of study participants. Participants were enrolled in the study in different ways depending on the preferences of the institutions that agreed to participate. For some institutions, the preference was to distribute the survey *via* institutional communication channels. Other institutions provided the research team with a list of email addresses, and these lists were used to distribute the survey *via* email. While anonymous survey links are desirable for one-time surveys, distribution of a more longitudinal survey *via* personalized links was found to reduce the overall email footprint and greatly enhance the ability to accurately link and track responses over time.

Although recent work has indicated that there is likely little to no effect of participation on wellbeing in COVID-19 survey research studies (Sollis et al., 2021), the research team was extremely sensitive to the potential impacts of study participation on participant wellbeing. Several strategies were implemented with a goal of balancing empirical rigor and issues of survey length and participation fatigue. First, the research team brainstormed all possible survey measures that capture constructs of interest. Then, in cases where all else was equal between two measures of a particular construct, the shorter measure was retained. The research team pilot tested the survey to determine approximate duration, aiming for no longer than 20 min. Finally, as the nature of the COVID-19 pandemic changed across the duration of the study, the research team implemented IRB-approved modifications to survey items to remove items that became less relevant and add items that became more relevant. For example, once the COVID-19 vaccine became available, items reflecting whether participants had received a vaccination were added. Similarly, the interview component of the study involved establishing a goal of 45 min or less to complete the interview; questions were developed to meet this goal, and questions were adapted to best reflect the stage of the pandemic at each time point.

Beyond participant enrollment and the structure of the survey and interview protocols, methods for retaining participants included a gift card research incentive and the distribution of personalized reports upon completion of the third and final survey. The research incentive provided a small compensation for participation ($3 for each survey completed plus an $11 bonus for completing all three surveys, for a total possible amount of $20). The personalized reports were designed in the survey platform and distributed automatically once the final survey was completed. They included individual scores (when available), ways to interpret those scores, and resources including mental health support.

Still, it was challenging to retain participants across the three time points at which data were collected. The first survey (Summer 2020) was sent to over 25,000 eligible faculty and students across all participating institutions. There were 2,935 surveys started and 1,015 complete surveys once careless responding screening was conducted. The second survey (Fall/Winter 2020) was distributed to 2,888 participants who consented to participate. There were 839 surveys started and 513 completed responses. A $11 incentive bonus was added to the third survey (Spring 2021) to promote retention. This time, there were 1,014 surveys started and 833 completed, indicating that the bonus improved response rates compared to the second survey.

Longitudinal research that examines individuals' experiences with disasters over time can make meaningfully advance our understanding of such disruptive situations; yet the challenges such research poses, including participant retention, make it difficult to conduct (McLeod et al., 2022). In this case, the research team originally planned for a significantly higher sample size than what was ultimately collected to support multilevel analyses. One lesson learned was the importance of mixed methods and multi-source data in human subjects-focused rapid research. In other words, given the potential fallibility of any one method in a rapid research environment, it is important to find ways within one's own research context to triangulate the ephemeral data of interest *via* multiple data collection mechanisms—each method addressing a potential limitation of another. Although some of the original analyses planned may not have had sufficient power to be conducted, the fundamental research questions of the project were still able to be answered given the richness of data provided by surveys, interviews, and institutional documents. Overall, the research team adapted to participant retention challenges by revising the survey and interview protocol to adjust to the changing landscape of the COVID-19 pandemic, using survey modifications to find a balance between empirical priorities and participant fatigue issues, and reorganizing funds to further incentivize participation in the final survey.

## Discussion

Ultimately, the value of insights gained from rapid research outweighs the challenges faced in conducting this research that responds with agility to emerging societal needs. The preceding content describes the challenges faced by a research team conducting rapid research on responses to the COVID-19 pandemic in institutions of higher education, the solutions used to respond to these challenges, and lessons learned that can potentially aid in future rapid research. Similar to recent work on rapid research during COVID-19 (Vindrola-Padros et al., 2020), the challenges described above are characterized by themes related to research partnerships and teamwork, study design and execution, and participant recruitment and retention. This paper adds to the existing literature on rapid research that has identified important challenges associated with adapting methods to the needs of the situation and in consideration of what the participants are going through while ensuring that the research questions at the core of the project are still addressed. Existing literature on challenges of conducting rapid research during times of crisis focuses primarily on health systems (e.g., Johnson and Vindrola-Padros, 2017) and qualitative methods (e.g., Rahman et al., 2021). This paper corroborates these previous findings from the perspective of a more quantitative-leaning, mixed-method study focused on higher education as a domain somewhat more distally affected by COVID-19 than public health or healthcare.

In this rapid research study, challenges arose in the logistics of coordination among a multi-institutional research team, in recruiting a large and representative sample of institutions, and in promoting participant retention. A common theme underlying these challenges seemed to be the human toll of the pandemic; the researchers' institutions, sampled institutions, and participants were all overburdened. In these situations, additional planning and coordination, anticipation of challenges, creativity in finding data sources that preserve the original aims of the project, and acknowledgment of the value institution and participant time allowed the research team to adapt. A summary of lessons learned and directions for future research corresponding to each of these challenges can be found in Table 1.

**Table 1:** Future rapid response human subjects research directions.

**Research challenge**	**Lessons learned**	**Questions for future research**
• Inter-institutional Research Team Coordination	• Use guidelines from the science of team science to inform research team formation and functioning • Promote research team norms of closed-loop communication and constant documentation • Hold data organization and cleaning meetings to develop shared mental models of data structure prior to analysis	• To what extent does existing infrastructure support vs. hinder collaborations across institutions in general and during rapid research in particular? • Where and through what means have institutions successfully implemented practices or policies that promote collaboration as much as individual scholarship? • How might disciplinary professional organizations support rapid research collaborations across institutions?
• Institutional Recruitment	• Create multiple data collection contingencies and backup plans • When relying on the participation of institutions facing crisis, over-sample representative populations if possible • Identify alternative sources of publicly available and archival institutional data	• To what extent would the existence of a centralized crisis and disaster-related institutional reporting database facilitate both practice and rapid response research? • What patterns exist in the ways in which institutions support their members' participation in rapid research and how does participation vs. non-participation affect the wellbeing of institutions and their members?
• Participant Retention	• Ensure study design is aligned with and supports goals for participant engagement in rapid research (e.g., minimize survey fatigue when studying distress during disasters) • Find ways to provide participants with as much benefit for participating in rapid research as the researchers benefit from their participation (e.g., provide customized feedback reports with score interpretation and wellbeing resources)	• What unobtrusive measures are best suited to supplement traditional survey and interview methods in rapid research, and what are their strengths and drawbacks? • What are the most ethical and effective mechanisms for sustaining motivation to remain engaged with rapid research across multiple time points? • What new and emerging technologies can be leveraged to reinvigorate participant recruitment and retention practices so as to meet the unique needs of rapid human subjects research?

Institutions are encouraged to engage in capacity building that reduces barriers to conducting rapid research, such as developing procedures that support inter-institutional collaboration ahead of time. We also urge researchers to engage in contingency planning early in the study design process, anticipate and offset the burden of participation as much as possible, and consider data sources that could compliment insights gained from participant report or supplement when obstacles interfere with original study plans. Finally, we encourage institutions and researchers to avoid becoming discouraged by the challenges of conducting rapid research. These efforts are critical; neglecting the voice of those affected would severely limit the ultimate impact of scientific research in times of crisis or disaster.

The experience and analysis described above has its limitations. The rapid research study was conducted within a higher education context that may not be applicable to all rapid research domains. We acknowledge that not all rapid research can or will be supported with funding to allow for components such as monetary participant incentives. The challenges and insights are derived from a study relying on primarily subjective, self-report survey methods and may not be applicable to studies with primarily objective measures. Finally, the primary focus on psychological constructs may limit its generalizability to other social sciences. Still, the hope in presenting these challenges and lessons learned is that others will still be able to derive insights that may enhance rapid research initiatives conducted with human subjects in the future.

## Data availability statement

The original contributions presented in the study are included in the article/supplementary material, further inquiries can be directed to the corresponding author.

## Ethics statement

Ethical review and approval was not required for the study on human participants in accordance with the local legislation and institutional requirements. Written informed consent from the [patients/ participants OR patients/participants legal guardian/next of kin] was not required to participate in this study in accordance with the national legislation and the institutional requirements.

## Author contributions

CL developed the outline and structure of the manuscript and contributed individual sections. KH and NS contributed individual sections to the manuscript. All authors contributed to the article and approved the submitted version.

## Funding

This material is based upon work supported by the National Science Foundation under Grant No. 2029754.

## Conflict of interest

The authors declare that the research was conducted in the absence of any commercial or financial relationships that could be construed as a potential conflict of interest.

## Publisher's note

All claims expressed in this article are solely those of the authors and do not necessarily represent those of their affiliated organizations, or those of the publisher, the editors and the reviewers. Any product that may be evaluated in this article, or claim that may be made by its manufacturer, is not guaranteed or endorsed by the publisher.

## Author disclaimer

Any opinions, findings, and conclusions or recommendations expressed in this material are those of the author(s) and do not necessarily reflect the views of the National Science Foundation. Work on this manuscript began while CL and KH held prior positions at Embry-Riddle Aeronautical University and the University of Central Florida, respectively.
